# The Effect of Urbanization on Ant Abundance and Diversity: A Temporal Examination of Factors Affecting Biodiversity

**DOI:** 10.1371/journal.pone.0041729

**Published:** 2012-08-02

**Authors:** Grzegorz Buczkowski, Douglas S. Richmond

**Affiliations:** Department of Entomology, Purdue University, West Lafayette, Indiana, United States of America; Stanford University, United States of America

## Abstract

Numerous studies have examined the effect of urbanization on species richness and most studies implicate urbanization as the major cause of biodiversity loss. However, no study has identified an explicit connection between urbanization and biodiversity loss as the impact of urbanization is typically inferred indirectly by comparing species diversity along urban-rural gradients at a single time point. A different approach is to focus on the temporal rather than the spatial aspect and perform “before and after” studies where species diversity is cataloged over time in the same sites. The current study examined changes in ant abundance and diversity associated with the conversion of natural habitats into urban habitats. Ant abundance and diversity were tracked in forested sites that became urbanized through construction and were examined at 3 time points - before, during, and after construction. On average, 4.3±1.2 unique species were detected in undisturbed plots prior to construction. Ant diversity decreased to 0.7±0.8 species in plots undergoing construction and 1.5±1.1 species in plots 1 year after construction was completed. With regard to species richness, urbanization resulted in the permanent loss of 17 of the 20 species initially present in the study plots. Recovery was slow and only 3 species were present right after construction was completed and 4 species were present 1 year after construction was completed. The second objective examined ant fauna recovery in developed residential lots based on time since construction, neighboring habitat quality, pesticide inputs, and the presence of invasive ants. Ant diversity was positively correlated with factors that promoted ecological recovery and negatively correlated with factors that promoted ecological degradation. Taken together, these results address a critical gap in our knowledge by characterizing the short- and long-term the effects of urbanization on the loss of ant biodiversity.

## Introduction

Urbanization is a major threat to biodiversity [1]–[5] and is responsible for species extinctions and biotic homogenization. The disturbance created by urbanization destroys the habitat of a wide array of unique endemic species and often creates an attractive habitat for relatively few species able to adapt to urban conditions [6]. This may lead to biotic homogenization whereby the genetic, taxonomic, or functional similarity of regional biota increases over time [7]–[8]. Emerging evidence suggests that biotic homogenization is occurring in a variety of ecosystems [4,9–10) with important ecological and evolutionary consequences [11]. As urbanization spreads rapidly across the globe, a key question for urban ecology and a basic challenge for conservation is to understand how it affects biodiversity [3].

Although urbanization provides excellent opportunities to test the effects of habitat alteration, degradation, and fragmentation on ecological communities, urbanizing landscapes have received relatively little attention with most research efforts being focused on more natural processes [12]–[14]. Studying ecological processes in urban environments is a relatively new direction in ecology [15]–[16]. To date, most studies have focused on birds [17]–[19] and we know much less about other vertebrates and very little about arthropod communities [20]. A recent review [19] of invertebrates from a variety of urbanized habitats reports that diversity decreased in 64% of studies, increased in 30% of studies, and remained unchanged in 6% of studies with the losses driven mostly by native species extinction and the gains by non-native species additions. Such variability in findings likely reflects the wide range of taxa and functional groups represented by the invertebrates.

Arthropods are excellent candidates for studying the effects of urbanization because they perform a wide range of ecosystem services and serve as important bioindicators of ecological change [19], [21]–[22]. Ants in particular are important because they represent a variety of trophic levels, have relatively short generation times and therefore respond quickly to environmental change, and they are important economic components of human-altered habitats [19], [20], [22]–[25]. Ants are a remarkable example of animals adapting to urban habitats [26]–[27] and the ecological and economic impacts of ants, especially invasive species, are well documented [28]–[29]. Ants are also abundant, highly diverse, and easy to collect and identify [30]–[32].

Numerous studies have examined the effect of urbanization on ant species richness [24], [33]–[39] and most studies implicate urbanization as the major cause of extinctions [28], [36], [40]. However, very few studies have identified an explicit connection between urbanization and biodiversity loss. Doing so requires long-term observations to document temporal changes in species inventories over time, and such data is logistically difficult to obtain and typically unavailable. As a result, the impact of urbanization is typically inferred indirectly by comparing species diversity along spatial gradients, typically by examining diversity along urban-rural gradients at a single time point [24], [27], [35], [41]. Urban-rural gradient studies are clearly a simplification of the complex patterns produced by urbanization [42]. A typical approach is to compare species abundance and diversity along gradients of urbanization (e.g. urban vs. rural areas, urban edge vs. inner city, urban green spaces vs. residential areas) and subsequently correlate species diversity and composition with habitat characteristics. Clearly, there are problems with this approach because the effect of urbanization is confounded by numerous extraneous factors and the evidence for the role of urbanization is only correlational rather than direct. For example, subtle differences in microhabitat characteristics between intact and urbanized sites might contribute to the observed differences in diversity making it difficult to isolate the role of urbanization. A different approach is to focus on the temporal rather than the spatial aspect and perform “before and after” studies where species diversity is carefully cataloged over time in the same sites and the role of habitat disturbance is examined directly over time.

The current study is a large-scale, long-term, survey-based examination of changes in ant abundance and diversity associated with the conversion of natural habitats into urban habitats. It represents a novel approach to studies on the effect of urbanization on native communities because it emphasizes the temporal component (i.e. comparing biodiversity in the same site before and after disturbance) rather than the spatial component (i.e. comparing biodiversity across disturbance gradients at a single time point). Therefore, it allows the opportunity to isolate the effect of urbanization on biodiversity without the confounding effects of other environmental factors. The study had two main objectives. The first objective was to track changes in ant abundance and diversity in forested sites that became urbanized through residential construction. Ant diversity was examined at 3 distinct time points - before, during, and after construction - with the prediction that biodiversity would decline as a result of disturbance. The second objective was to examine the recovery of ant fauna in developed residential lots based on several factors such as time since construction, neighboring habitat quality, pesticide inputs, and the presence of dominant, invasive ant species. The prediction was that ant diversity would be positively correlated with factors that promote ecological recovery (e.g. proximity to undisturbed sites) and negatively correlated with factors that promote ecological degradation (presence of invasive ant species, pesticide inputs). Taken together, the results of these two objectives address a critical gap in our knowledge by investigating how urbanization affects the richness and abundance of ants.

## Methods

### Study Sites and Research Plots

The study was carried out in The Orchard, a 94 acre (38 ha) residential development site centered at 40.44°N and 86.95°W in West Lafayette, Indiana, U.S.A. The Orchard is an abandoned apple orchard where commercial apple production ceased approximately 20 years ago and residential development begun approximately 10 years ago. The Orchard is successionally advanced with dense shrub understory (dominant species include bush honeysuckle, *Lonicera spp.* and autumn olive, *Elaeagnus umbellata*), thick herbaceous ground cover, and numerous hardwood trees overtopping the naturalized apple trees. Old, abandoned apple orchards provide extremely important habitat to a myriad of species that require early successional habitat, especially insects, birds, reptiles, and small mammals [43]. The site is comprised of approximately 145 lots that are available for individual purchase prior to construction. The majority of lots are approximately 1,000 square meters (0.25 acres) in size. Developed lots consist of housing of various age constructed within the last 10 years. These houses are interspersed among undeveloped orchard lots. Prior to construction, the lots are cleared of all vegetation (with the exception of a few desirable hardwood saplings) and the topsoil is removed. After construction, the landscaping is installed consisting mainly of sodded lawn, mulched flower beds, and additional landscape trees.

### Effect of House Construction on Ant Abundance and Diversity

**Figure 1:**
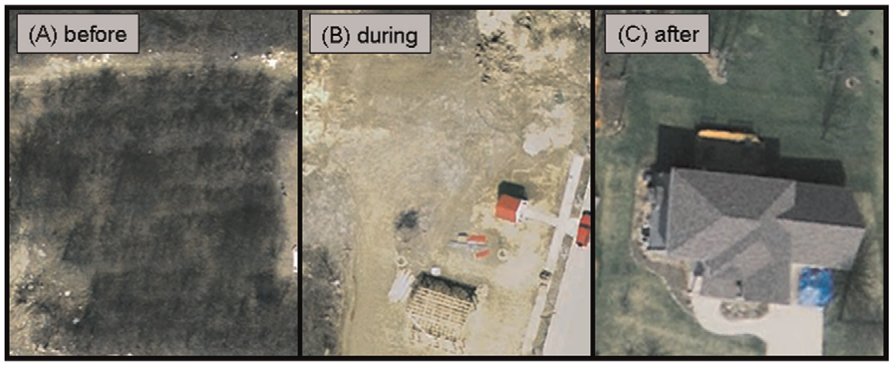
Aerial photos of research plots representative of each stage of habitat disturbance. (A) before construction: naturalized apple orchard, (B) during construction: trees and top soil are removed to prepare the ground for construction, (C) after construction: sodded lawn and landscape trees are installed, the house covers majority of the plot.

The impact of house construction activities on ant abundance and diversity was examined in 15 lots throughout The Orchard. For each lot, ant abundance and diversity were estimated at 3 time points: before, during, and after construction (Fig. 1). The houses were sampled approximately 6–12 months prior to construction, during construction (typically within 1–2 weeks after construction begun), and 1 year after construction was completed. A combination of baiting and visual searching was used at all lots to estimate ant abundance and diversity. Ten note cards baited with a blend of peanut butter and corn syrup (50∶50, v:v) [44] were placed on the ground in each lot. Prior to construction, the cards were placed uniformly throughout the plot. After construction, the cards were placed around the foundation of the house and throughout the yard. The bait cards were collected 2 hours after placement, placed in individual plastic bags, and the ants were later identified to species in the lab. In addition to baiting, visual searches were conducted throughout the sites. This involved turning over rocks and logs, inspecting debris on the ground, and looking for signs of ant activity on the ground. The searching effort was standardized across plots by having 2 people sample each plot for approximately 15 minutes. Ant abundance (the percentage of bait stations that had at least 1 ant on it) and ant diversity (the total number of ant species present) were then calculated for each site.

### Ant Fauna Recovery in Residential Lots

The long-term recovery of ant fauna in developed lots was examined by sampling ant abundance and diversity at houses of various age. The objective was to examine the relationship between house age (years since construction) and ant abundance and diversity. The sampling procedure was as above, with 10 bait cards per lot. In total, 51 houses were sampled. All houses were single family dwellings with traditional landscaping that included ornamental plants around the foundation, mulched patches of shrubs or trees not adjacent to the house, and a mowed lawn covering majority of the yard. In addition, the importance of various factors that could potentially affect ant communities was investigated. The homeowners were surveyed regarding pesticide use around homes to determine the potential effect of chemical insect control on ant presence. They were asked whether any ant control products had been used on the property in the last 3 years and responses were recorded as either yes (1) or no (0). In addition, the type of property bordering the sampled houses on either side was recorded. The surrounding lots were categorized either as developed (another house) or undeveloped (orchard). Undeveloped orchard lots serve as refugia for a variety of ant species and could potentially serve as a source of ants for nearby developed lots. For data analysis, houses surrounded by two undeveloped lots received a value of 0, surrounded by 1 developed lot and 1 undeveloped lot a value of 1, and surrounded by 2 developed lots a value of 2.

### Statistical Analysis

The effect of house construction on ant abundance and diversity was estimated by using an ANOVA test (PROC GLM procedure) in SAS 9.2 [45] with time (before, during, after) as an independent variable and abundance or diversity as dependent variables. The ANOVA analyses were followed by post-hoc Tukey’s HSD tests to separate the means. The relationship between house age and the percentage of developed adjacent lots was examined using simple linear regression. A homogeneity of slopes ANCOVA model was used to determine if the relationship between house age and ant abundance and diversity parameters varied depending on the history of insecticide use around the structure and a separate slopes ANCOVA model was then used to describe the relationship between house age and ant abundance and diversity parameters separately according to insecticide history.

## Results

### Effect of House Construction on Ant Abundance and Diversity

**Figure 2:**
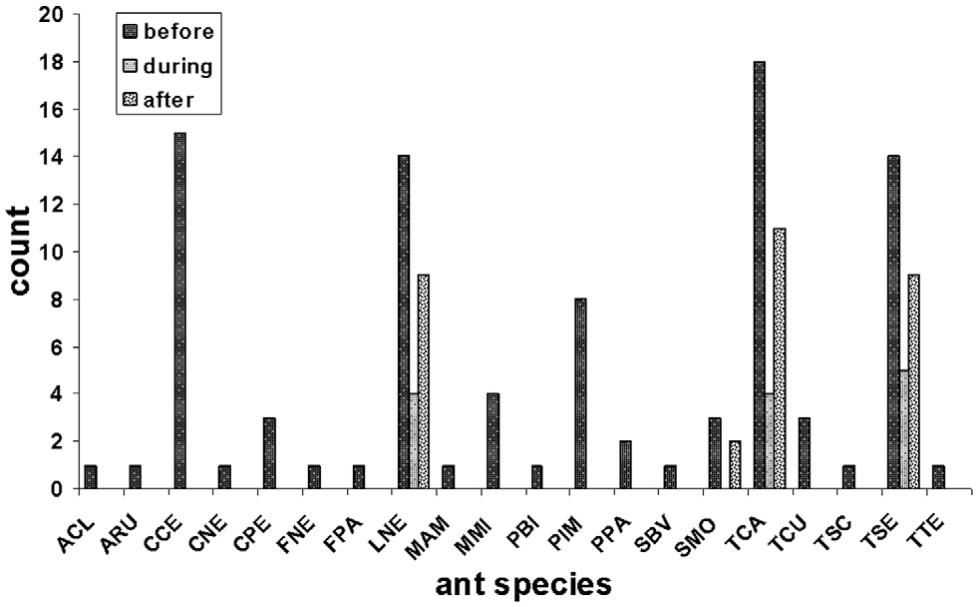
Ant species abundance before, during, and after construction. For each time category, count is the total number of baits stations where a given species was detected. In total, 20 species were detected in the study: ACL (*Acanthomyops claviger*), ARU (*Aphaenogaster rudis*), CCE (*Crematogaster cerasi*), CNE (*Camponotus nearcticus*), CPE (*Camponotus pennsylvanicus*), FNE (*Formica neogagates*), FPA (*Formica palleidefulva*), LNE (*Lasius neoniger*), MAM (*Myrmica americana*), MMI (*Monomorium minimum*), PBI (*Pheidole bicarinata*), PIM (*Prenolepis imparis*), PPA (*Paratrechina parvula*), SBV (*Stenamma brevicorne*), SMO (*Solenopsis molesta*), TCA (*Tetramorium caespitum*), TCU (*Temnothorax curvispinosus*), TSC (*Temnothorax schaumii*), TSE (*Tapinoma sessile*), TTE (*Temnothorax texanus*).

The degree of environmental disturbance (before, during, or after construction) had a significant effect on ant abundance (ANOVA, *F* = 48.35, df = 2, *P*<0.0001). In undisturbed orchard plots, the ants were present on 6.3±2.0 bait stations. In contrast, the ants were present on only 0.9±1.1 bait stations in plots undergoing construction (85% decline; t test, *t* = 9.81, df = 28, *P*<0.0001) and 2.1±1.4 bait stations in plots 1 year after the completion of construction (64% decline; t test, *t* = 9.81, df = 28, *P*<0.0001). The degree of environmental disturbance also had a significant effect on ant diversity (ANOVA, *F* = 50.86, df = 2, *P*<0.0001). On average, 4.3±1.2 unique species were detected in undisturbed orchard plots prior to construction (Table 1). Ant diversity decreased to only 0.7±0.8 species in plots undergoing construction (84% decline; t test, *t* = 9.81, df = 28, *P*<0.0001) and 1.5±1.1 species in plots 1 year after the completion of construction (63% decline; t test, *t* = 9.81, df = 28, *P*<0.0001). With regard to species richness (*S*), a total of 20 ant species were detected in undisturbed orchard plots (Table 1, Figure 2). Construction activities resulted in the permanent loss of 17 species (85% decline) and only 3 species were present right after construction was completed and 4 species were present 1 year after construction was completed. No statistical difference was detected in ant abundance or diversity between experimental plots during and after construction (Table 1). Species identity for the ants discovered in the experimental plots is shown in Figure 2. Of the 20 species detected in undisturbed plots, 3 were relatively abundant prior to construction, persisted during construction, and experienced a relatively fast recovery: *Lasius neoniger* (LNE), *Tetramorium caespitum* (TCA), and *Tapinoma sessile* (TSE). Other species, such as *Crematogaster cerasi* (CCE) or *Prenolepis imparis* (PIM) were relatively common prior to disturbance, but were unable to recover once construction was completed and were absent from the plots. Still, other species such as *Solenopsis molesta* (SMO) were relatively rare, but were able to persist and recover.

**Table 1:** Ant faunal diversity in plots before, during, and after construction.

sampling time	ant abundance^1^	ant diversity^2^	*S* 3	*D* 4	*H’* 5	*J’* 6
before construction	6.3±2.0 a	4.3±1.2 a	20 a	0.11	1.05	0.81
during construction	0.9±1.1 b	0.7±0.8 b	3 b	0.26	0.47	0.99
after construction	2.1±1.4 b	1.5±1.1 b	4 b	0.27	0.55	0.91

1 Mean (± SD) number of bait stations with ants present (out of 10 stations, averaged over 15 plots).

2 Mean (± SD) number of ant species discovered (averaged over 15 plots).

3 Ant species richness (the total number of ant species discovered in experimental plots).

4 Simpson index.

5 Ant species diversity (Shannon index).

6 Ant species equitability.

Numbers within columns followed by the same letter are not different based on a Tukey test (*P* = 0.05).

### Ant Fauna Recovery in Residential Lots

**Figure 3:**
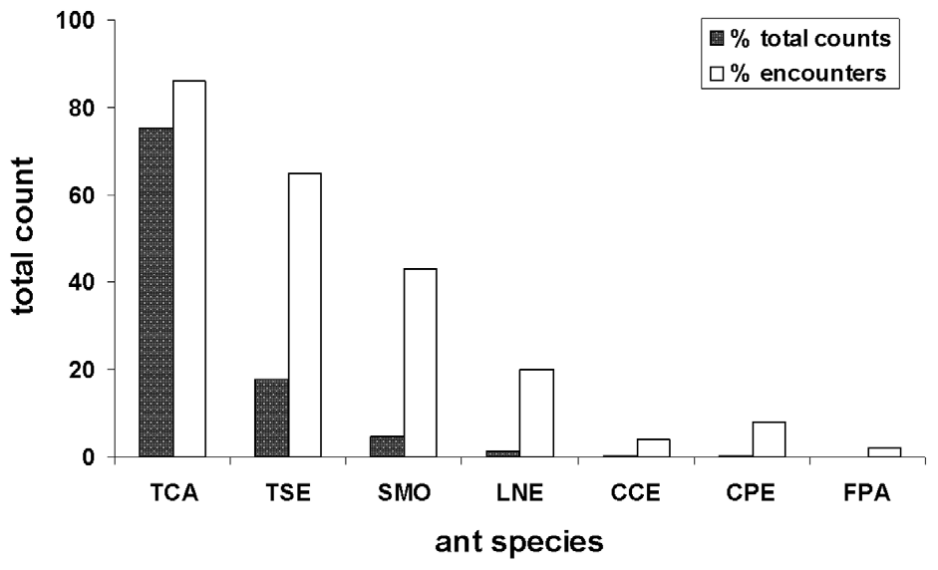
The relative abundance of the seven ant species found in post-construction plots. (A) relative abundance expressed as the percentage of the total number of ants collected at the bait stations, (B) relative abundance expressed as the percentage of the homes where each species was encountered. In both (A) and (B), *n* = 510 bait stations; 51 houses with 10 bait stations per house. Species names as in Figure 2.

**Figure 4:**
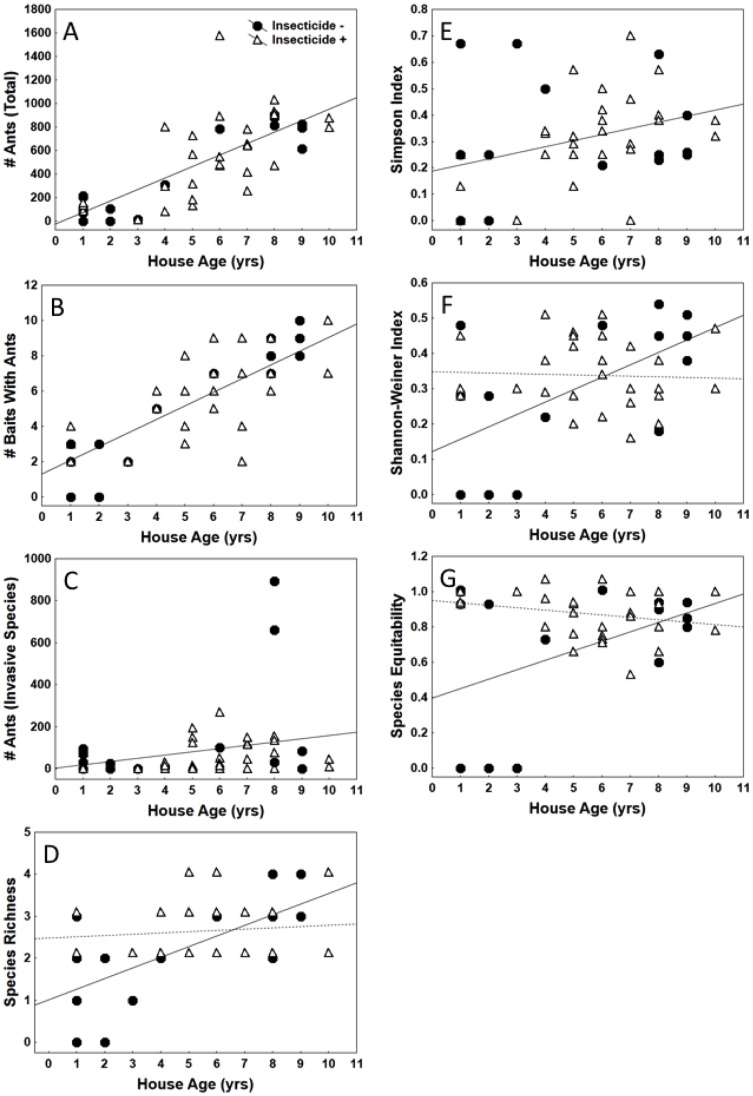
The relationship between house age and various metrics of ant diversity and abundance. (A) the total number of ants discovered, (B) the number of bait stations with ants present (C) the total number of invasive ants discovered, (D) ant species richness, (E) ant species diversity (Simpson), (F) ant species diversity (Shannon-Weiner) and (G) ant species equitability at each site. When two regression lines are present, separate models were necessary based on history of insecticide use.

Ant communities were sampled around 51 houses of varying age and a total of 22,560 ants belonging to 7 species were detected (Figure 3). The ants were present on 116 out of 510 (23%) bait stations placed around the houses. Ant activity, as indicated by the number of bait stations with ants present, ranged between 0 and 10 (out of 10 bait stations) and averaged 5.0±2.8 baits per house. The number of species per house ranged between 0 and 4 and averaged 2.3±1.0 species. Pavement ants, *Tetramorium caespitum* (TCA) dominated the counts (Figure 3). They comprised 75% of all ants encountered at the bait stations and were present at 44/51 (85%) of the houses. Odorous house ants, *Tapinoma sessile* (TSE) were the second most frequently encountered ant. They comprised 18% of all ants encountered at the bait stations and were present at 33/51 (65%) of the houses. The remaining 5 species accounted for the remaining 7% of the ants. A significant correlation was detected between house age and ant abundance (Figure 4A, Pearson’s correlation, *r* = 0.79, *P*<0.0001) suggesting that ant counts increase around older houses. However, this increased abundance is mainly due to high numerical presence of a few species able to persist in urban environments, not high species diversity. Likewise, a significant correlation was detected between house age and ant diversity (Fig. 4B, Pearson’s correlation, *r* = 0.51, *P* = 0.0001) and between house age and the number of baits with ants present (Fig. 4C, Pearson’s correlation, *r* = 0.82, *P*<0.0001). No significant relationship was detected between insecticide use and the total ant count around homes (ANCOVA, *F* = 0.02, df = 1, *P* = 0.894). Of the 51 houses that participated in the study, 21 (41%) had used some form of outdoor pest control in the last 3 years, and 30 (59%) did not (t test, *t* = −1.85, df = 49, *P* = 0.071). Interestingly, the average number of ants found around homes that used pesticides, 520±364, was higher than the number of ants found around homes that did not, 331±354, although not significantly (t test, *t* = 2.01, df = 44, *P* = 0.070). A significant negative correlation was detected between the total number of *T. caespitum* and the total number of all other ant species present around the houses (Pearson’s correlation, *r* = 0.79, *P*<0.0001) suggesting that *T. caespitum* may negatively affect native ant diversity in urban environments. Proximity of developed lots to undisturbed lots did not. Of the 51 houses included in the study, 10 (20%) were surrounded by 2 undeveloped lots (307±343 ants present), 16 (31%) were surrounded by 1 developed lot and 1 undeveloped lot (271±314 ants present), and 25 (49%) were surrounded by 2 developed lots (606±350 ants present).

### Effect of House Age on Ant Abundance and Diversity

As house age increased, so did the probability that adjacent lots were developed (*F* = 15.1, df = 1, 49, *P* = 0.0003). The influence of house age on ant abundance and diversity varied according to the history of insecticide use around the structure (*F* = 1.8, df = 1, 41, *P* = 0.11) with significant interactions between house age and insecticide use observed for the total number of ant species recorded, H, and J (Table 2). On properties with no recent history of insecticide use, the total number of ant species, H, and J all increased as house age increased (Table 3, Fig. 4). On properties with a recent history of insecticide use, there was no significant relationship between house age and any of these parameters. Total ant numbers, total invasive ant numbers, total ants at bait stations, and D all increased significantly with house age regardless of insecticide use.

**Table 2:** F-statistics and degrees of freedom (df) for a homogeneity of slopes model used to examine the relationship between house age and ant abundance and diversity parameters as a function of the history of insecticide use around the structure.

factor	df	total^1^	baits^2^	invasive^3^	*S*	*D*	*H’*	*J’*
insecticide	1, 47	0.1	3.1	0.1	8.4**	0.6	9.1**	12.1**
house age	1, 47	66.1****	83.6****	5.9*	11.7**	6.5*	6.1*	2.0
insecticide×house age	1, 47	0.0	4.0	2.1	7.1*	0.7	7.5**	5.6*

* P≤0.05,

** ≤P 0.01,

*** ≤P 0.001,

**** P≤0.0001.

1 the total number of ants discovered at 10 bait stations placed around the house.

2 the total number of bait stations with ants present (x/10).

3 the total number of pavement ants, *Tetramorium caespitum*, discovered at 10 bait stations placed around the house.

ant diversity parameters as in Table 1.

**Table 3:** Mean responses, parameter estimates and performance statistics for separate slopes models used to describe the influence of structure age and insecticide use around the structure on the density and composition of the ant community in a rural housing development established in a former apple orchard.

responsevariable^1^	insecticideusage	mean (±SE)	parameter estimate (±SE)	t	p	R^2^
total	NA	442.16±51.61	97.68±11.01	8.87	<0.000001	0.62
baits	NA	5.00±0.39	0.77±0.08	9.79	<0.000001	0.69
invasive	NA	78.14±21.99	15.49±7.25	2.13	0.037523	0.18
*S*	No	1.95±0.28	0.25±0.06	4.60	0.000032	0.37
*S*	Yes	2.53±0.12	0.03±0.06	0.50	0.616778	–
*D*	NA	0.30±0.03	0.02±0.01	2.62	0.011791	0.14
*H’*	No	0.25±0.04	0.04±0.01	3.94	0.000270	0.31
*H’*	Yes	0.34±0.02	−0.01±0.01	−0.18	0.855200	–
*J’*	No	0.60±0.10	0.05±0.02	2.85	0.006394	0.31
*J’*	Yes	0.87±0.02	−0.01±0.02	−0.63	0.529624	–

* NA = not applicable as determined by homogeneity of slopes ANCOVA.

1 response variables as in tables 1 and 2.

## Discussion

This study represents a novel approach to studies on the effect of urbanization on native communities because it emphasizes the temporal component (i.e. comparing biodiversity in the same site before and after disturbance) rather than the spatial component (i.e. comparing biodiversity across disturbance gradients at a single time point). Previous studies were largely a simplification of the complex patterns produced by urbanization [19], [42], [46] because they largely failed to account for any climatic, geographic, historical, or spatial scale factors that were unique to each site. Habitat and landscape factors are known to be important determinants of ant communities [23], [38]–[39], [47]–[49] and comparisons of different sites along urban gradients carry a significant bias. The current study allowed a unique opportunity to document the process of urbanization through time at a single location, avoiding the potentially confounding effects of other location-related factors associated with many similar experiments. Biodiversity was tracked in natural sites that subsequently experienced urban disturbance and a profound effect of urbanization was discovered. Urbanization resulted in the permanent loss of 17 of the 20 species initially present in the study plots and recovery was slow as indicated by the lack of significant improvement in species richness 1 year after construction was completed. Environmental disturbance had a severely negative effect on ant abundance and diversity which declined by 85% and 84%, respectively. Species richness also experienced a significant decline. This suggests that previous studies, which focused mainly on the spatial component and discovered relatively minor diversity losses, may have underestimated the impact of urbanization.

Urbanization creates intensively managed, homogenous landscapes and forces native species that adapt to a relatively uniform environment that is often radically different from the undeveloped habitat. Under such scenario, many ecological specialists become locally extinct and are replaced by a few ecological generalists that are broadly adapted and able to tolerate or even benefit from human activity [6]. This may lead to biotic homogenization, where the rapid and drastic environmental change promotes the geographic reduction of some species (‘losers’) and the geographic expansion of others (‘winners’) [6]. In the current study the ‘losers’ were species with relatively sensitive nesting and/or feeding requirements that were unable to tolerate disturbance. The ‘winners’ were typically disturbance specialists that were able to tolerate disturbance and recover fairly quickly. House construction created a highly uniform disturbance where all lots were cleared of trees and topsoil. Previous results show that this type of disturbance has the greatest effect on epigeic ant species which utilize above-ground organic debris as nesting and feeding sites, and the lowest effect on hypogaeic ant species which have subterranean nests [38]. Arboreal species such as *Crematogaster cerasi* (CCE) and *Camponotus pennsylvanicus* (CPE) and cavity-nesting species such as *Temnothorax curvispinosus* (TCU) and *Monomorium minimum* (MMI) were fairly common prior to disturbance and completely absent following disturbance. In contrast, subterranean species such as *Lasius neoniger* (LNE), *Solenopsis molesta* (SMO), and *Tetramorium caespitum* (TCA) appeared largely unaffected by the disturbance. In fact, *L. neoniger* and *T. caespitum* were frequently observed rebuilding their nests in heavily compacted, clayey subsoil soon after the lots were cleared.

Of the 20 species detected in undisturbed plots, 3 were relatively abundant before, during, and after development: cornfield ants (*Lasius neoniger*), pavement ants (*Tetramorium caespitum*), and odorous house ants (*Tapinoma sessile*). *Lasius neoniger* is the dominant open habitat species in the northeastern United States [50]–[51] and is common in urban areas [37], [44]. It is probably best classified as an urban adapter – a species that can adapt to urban habitats, but also utilizes more natural environments. The majority of the colonies nested in turf and did not seem to be closely associated with the structures themselves.

*Tetramorium caespitum* is an introduced species that has spread widely across the United States and is almost invariably associated with human disturbed sites [40], [52], [53], [54]. In a study by [37], *T. caespitum* comprised 53% of all ants collected in a highly urbanized habitat and they were the most abundant species in an ant survey conducted in West Lafayette, Indiana, approximately 2 km from the present study site [54]. In addition, [35] reported that ant richness in urban sites negatively correlated with the abundance of *T. caespitum* and [37] reported that *T. caespitum* abundance correlated negatively with tree density, indicating this species’ preference for open, disturbed sites.

*Tapinoma sessile* is widespread throughout North America and has the widest geographic range and greatest ecological tolerance of any ant in North America [55]. It is very opportunistic and inhabits a variety of nesting sites, both natural and man-made and in urban areas it is classified a pest species [56]. Recent work demonstrated that *T. sessile* is a highly plastic species with a flexible social structure [38], [57]. In natural habitats, *T. sessile* is a subdominant species comprised of small, single-queen colonies. In urban areas, *T. sessile* exhibits the characteristics common to most invasive ant species such as extreme polygyny (thousands of queens), extensive polydomy (multiple nests), and ecological dominance over native ant species [38], [57]–[59]. Furthermore, *T. sessile* has been recently reported as an invasive species in human-altered habitats in Hawaii [44]. Both *T. caespitum* and *T. sessile* are best categorized as urban exploiters or species that become dependent on humans for food and shelter [59]–[60]. The great majority of the colonies nested in mulch beds around the foundation of the house and under concrete pathways associated with the house. The ability to exploit abundant resource subsidies offered by humans was likely the primary reason for *T. caespitum* and *T. sessile* attaining such high population densities on converted sites.

Urbanization affected species richness in a variety of ways. The majority of ant species, especially those that nested in above-ground material, were physically removed from the site when the tree cover and the topsoil were removed. Urbanization also affected species richness through the species-area effect: the negative relationship between the area of a habitat and the number of species found within that area. In all urban habitats, large expanses of impervious concrete and asphalt pavement reduce and fragment the area available for life to survive. The typical size of a residential plot in this study was approximately 1,000 square meters. A typical footprint for the house, including concrete driveway and sidewalks, was approximately 350 square meters. Therefore, the area available for nesting and foraging was reduced by approximately 35%.

Another negative impact on biodiversity is related to the severe structural simplification of vegetation in urbanized areas. Trees serve as important nesting sites for many ant species and trees colonized by honeydew-producing hemipterans provide important feeding sites for many species. During urban development, mature trees were removed and replaced with various landscaping plants once construction was completed. The remaining area was covered by a monoculture of grass to create lawns. Previous studies show that the percentage canopy cover is an important factor influencing ant species richness [37]–[38], [41], [61]. A study by [24] demonstrated that land development can significantly affect ant diversity, even in areas that retain a substantial component of native vegetation. Land development and disturbance of 30–40% appeared to be the level above which ant diversity began to decline.

The second objective examined the recovery of ant fauna in developed residential lots based on several factors such as time since construction, neighboring habitat quality, and pesticide inputs, and the presence of dominant, invasive ant species (*Tetramorium caespitum*). The prediction was that ant diversity would be positively correlated with factors that promote ecological recovery (e.g. longer post-recovery time and proximity to undisturbed sites) and negatively correlated with factors that promote ecological degradation (e.g. pesticide use and the presence of invasives).

A significant relationship was discovered between house age (time since construction) and various metrics of ant presence (the total number of ants discovered on the bait stations, number of bait stations occupied by ants). House age is an important factor driving ant abundance as older houses have had more opportunities for colonization events to occur, either via the influx of ants from neighboring plots or the arrival of alate queens from more distant areas. Older houses typically have more mature landscaping which may provide the ants with various ecological niches not available around newly constructed homes. However, older houses were also more likely to be surrounded by developed (built-on) lots, making the possibility of emigration from adjacent undisturbed patches less likely. Not surprisingly, ants largely experienced a numerical recovery over time (as indicated by the total number of ants present on the bait stations), rather than a rebound in species richness. Older houses had significantly more ants relative to newer houses, but the increase was driven mostly by the presence of the invasive *T. caespitum*. In contrast, species richness never recovered and only 7 species were found around older construction (average 2.3±1.0 species per house; range 0–4), substantially lower than the 20 species present in undisturbed plots.

Houses categorized as affected by insecticide use were those that received some type of outdoor insecticide application in the last 3 years. Insecticides commonly used in such cases may range from neonicotinyls and phenyl pyrazoles with relatively long-term residual activity, to pyrethroids and organophosphates with much shorter persistence. As a result, the long-term effects of such treatments can vary widely [62]. Even targeted ant control treatments around structures typically do not result in complete elimination of all colonies, but rather offer temporary suppression [54]. In some cases, insecticide treatments may actually create empty ecological niches that are then filled by invading ant species previously absent from the sites [54]. Not surprisingly, insecticide use had mixed effects on ant abundance and diversity following the establishment of a structure.

While ant abundance generally increased with house age, ant diversity (Shannon), species richness, and equitability increased with house age only at locations without a history of insecticide use. These parameters remained flat over time at locations where insecticides were recently used. However, the likelihood of future increases in ant diversity, species richness and species equitability at insecticide free sites remains questionable due to the relatively high impact of just a few data points on the regression line for this class. The positive relationship between house age, and these indices appeared to be driven largely by three or four younger structures (<3 yrs old) registering zero or very low values compared to their insecticide-treated counterparts. Although any number of factors including insecticide usage on adjacent lots, misapplication of insecticides by homeowners, or the failure of ants to recolonize these particular sites for an extended period of time following disturbance could potentially explain this pattern, such speculation is beyond the scope of the current study. Further data collection aimed at clarifying a potential mechanism underlying these observations will be required. As such, the current study provides strong evidence that although ant abundance may recover within a few years after urbanization, gains are likely to be driven by a relatively few pest/invasive species. Recovery of ant species richness and diversity to pre-disturbance levels appears to be highly unlikely under current post-development landscape management regimes.
